# Supplementary figures and images for: Blocking Gi/o-Coupled Signaling Eradicates Cancer Stem Cells and Sensitizes Breast Tumors to HER2-Targeted Therapies to Inhibit Tumor Relapse
Source: Cancers (Basel). 2022 Mar 28;14(7):1719. doi: 10.3390/cancers14071719 (PMC8997047; doi:10.3390/cancers14071719)

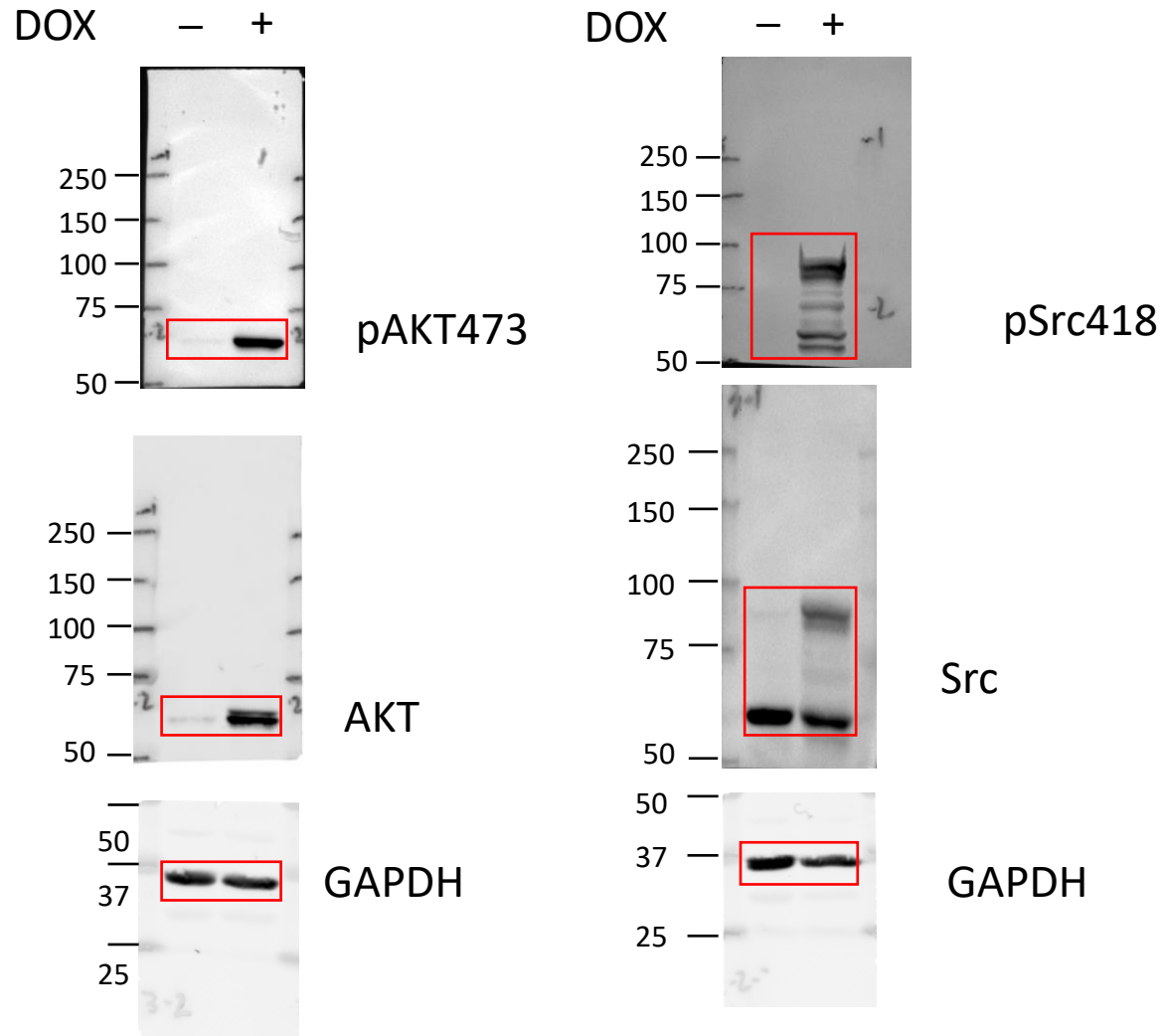

Supplemental Figure S1: The original blots for Figure 5E. The cropped bands are indicated by boxes.

Supplement: Supplementary file 1 [file cancers-14-01719-s001.zip › cancers-1644718-supplementary.pdf]
